# A Genome-Wide Investigation of MicroRNA Expression Identifies Biologically-Meaningful MicroRNAs That Distinguish between High-Risk and Low-Risk Intraductal Papillary Mucinous Neoplasms of the Pancreas

**DOI:** 10.1371/journal.pone.0116869

**Published:** 2015-01-21

**Authors:** Jennifer Permuth-Wey, Y. Ann Chen, Kate Fisher, Susan McCarthy, Xiaotao Qu, Mark C. Lloyd, Agnieszka Kasprzak, Michelle Fournier, Vonetta L. Williams, Kavita M. Ghia, Sean J. Yoder, Laura Hall, Christina Georgeades, Funmilayo Olaoye, Kazim Husain, Gregory M. Springett, Dung-Tsa Chen, Timothy Yeatman, Barbara Ann Centeno, Jason Klapman, Domenico Coppola, Mokenge Malafa

**Affiliations:** 1 Department of Cancer Epidemiology, H. Lee Moffitt Cancer Center, Tampa, FL, United States of America; 2 Department of Biostatistics and Bioinformatics, H. Lee Moffitt Cancer Center, Tampa, FL, United States of America; 3 Department of Clinical Testing Development, H. Lee Moffitt Cancer Center, Tampa, FL, United States of America; 4 Department of Analytic Microscopy, H. Lee Moffitt Cancer Center, Tampa, FL, United States of America; 5 Department of Tissue Core Administration, H. Lee Moffitt Cancer Center, Tampa, FL, United States of America; 6 Department of Information Shared Services, H. Lee Moffitt Cancer Center, Tampa, FL, United States of America; 7 Department of Molecular Genomics, H. Lee Moffitt Cancer Center, Tampa, FL, United States of America; 8 Department of Gastrointestinal Oncology, H. Lee Moffitt Cancer Center, Tampa, FL, United States of America; 9 Department of Anatomic Pathology, H. Lee Moffitt Cancer Center, Tampa, FL, United States of America; 10 Department of Gastroenterology, H. Lee Moffitt Cancer Center, Tampa, FL, United States of America; 11 Department of Gastrointestinal Surgical Oncology, H. Lee Moffitt Cancer Center, Tampa, FL, United States of America; 12 Department of Surgery, Gibbs Cancer Center and Research Institute, Spartanburg, SC, United States of America; University of Massachusetts Medical, UNITED STATES

## Abstract

**Background:**

Intraductal papillary mucinous neoplasms (IPMNs) are pancreatic ductal adenocarcinoma (PDAC) precursors. Differentiating between high-risk IPMNs that warrant surgical resection and low-risk IPMNs that can be monitored is a significant clinical problem, and we sought to discover a panel of mi(cro)RNAs that accurately classify IPMN risk status.

**Methodology/Principal Findings:**

In a discovery phase, genome-wide miRNA expression profiling was performed on 28 surgically-resected, pathologically-confirmed IPMNs (19 high-risk, 9 low-risk) using Taqman MicroRNA Arrays. A validation phase was performed in 21 independent IPMNs (13 high-risk, 8 low-risk). We also explored associations between miRNA expression level and various clinical and pathological factors and examined genes and pathways regulated by the identified miRNAs by integrating data from bioinformatic analyses and microarray analysis of miRNA gene targets. Six miRNAs (miR-100, miR-99b, miR-99a, miR-342-3p, miR-126, miR-130a) were down-regulated in high-risk versus low-risk IPMNs and distinguished between groups (*P*<10^−3^, area underneath the curve (AUC) = 87%). The same trend was observed in the validation phase (AUC = 74%). Low miR-99b expression was associated with main pancreatic duct involvement (*P* = 0.021), and serum albumin levels were positively correlated with miR-99a (r = 0.52, *P* = 0.004) and miR-100 expression (r = 0.49, *P* = 0.008). Literature, validated miRNA:target gene interactions, and pathway enrichment analysis supported the candidate miRNAs as tumor suppressors and regulators of PDAC development. Microarray analysis revealed that oncogenic targets of miR-130a (*ATG2B, MEOX2*), miR-342-3p (*DNMT1*), and miR-126 (*IRS-1*) were up-regulated in high- versus low-risk IPMNs (*P*<0.10).

**Conclusions:**

This pilot study highlights miRNAs that may aid in preoperative risk stratification of IPMNs and provides novel insights into miRNA-mediated progression to pancreatic malignancy. The miRNAs identified here and in other recent investigations warrant evaluation in biofluids in a well-powered prospective cohort of individuals newly-diagnosed with IPMNs and other pancreatic cysts and those at increased genetic risk for these lesions.

## Introduction

Pancreatic ductal adenocarcinoma (PDAC) is the fourth leading cause of cancer mortality in the United States, claiming the lives of nearly 40,000 individuals each year [1]. Surgical resection offers the best chance for improved survival, but 80–85% of cases are unresectable at diagnosis [1]. These statistics underscore the urgent need to develop strategies to detect PDAC at an early, operable stage. It is established that PDAC does not arise *de novo*, but instead marks the end of progression from one of three types of non-invasive precursor lesions arising within pancreatic ducts: pancreatic intraepithelial neoplasia (PanIN), mucinous cystic neoplasms (MCNs), and intraductal papillary mucinous neoplasms (IPMNs) [2]. While PanINs are microscopic lesions in ducts < 5 mm in diameter, MCNs and IPMNs are macroscopic mucinous cysts accounting for over half of the estimated 150,000 asymptomatic pancreatic cysts detected incidentally in the general population each year due to increased computed tomography and magnetic resonance imaging [3,4]. IPMNs have also been detected among those at high genetic risk for PDAC [5,6]. Although improvements in imaging, cytology, and molecular studies have enabled proper classification and management of some benign non-neoplastic pancreatic cysts, mucinous cysts such as IPMNs are challenging to manage due to the inability to accurately predict which lesions can be monitored, which are likely to progress to invasion, and which may have an associated invasive component [7]. Since data highlight a two-decade window of opportunity for early detection efforts in PDAC [8], IPMNs represent prime targets for early detection and prevention of progression to invasive, fatal disease.

IPMNs present within the main pancreatic duct (MD-IPMN), side branch ducts (BD-IPMN), or both (mixed-IPMN), and are further classified based on the degree of dysplasia which ranges from adenoma (low-grade dysplasia, LG) and borderline (moderate-grade dysplasia, MG) to carcinoma in situ (high-grade dysplasia, HG) and invasive carcinoma [2] (S1 Fig.). MD-IPMNs are associated with a higher grade and faster growth compared to BD-IPMNs, with the 5-year risk of developing HG or invasive disease from an adenoma to be ~ 63% for MD-IPMNs and 15% for BD-IPMNs [9]. Other predictors of malignant potential include main duct dilation (≥5mm), mural nodules, cyst size (≥3 cm), and symptoms such as jaundice and abdominal pain [3,10]. Consensus guidelines [10] recommend resection for surgically-fit patients with MD-IPMNs and careful observation for asymptomatic BD-IPMNs measuring <3 cm in the absence of mural nodules, main-duct dilation, or positive cytology. However, these guidelines do not reliably predict the degree of dysplasia pre-operatively [11–14]. To date, the only way to treat IPMNs and accurately identify the grade of dysplasia is through surgical resection and pathological evaluation, but risks of morbidity (ie. long-term diabetes) and mortality associated with a Whipple procedure or a distal or total pancreatectomy may outweigh the benefits, especially for patients with low-grade disease [15]. Alternatively, taking a ‘watch and wait’ approach could lead to a missed opportunity to cure a patient with occult invasive disease [15].

Although many DNA-, RNA- and protein-based markers are under investigation as markers of early pancreatic neoplasia, most require further validation [16]. MicroRNAs (miRNAs) are small non-coding RNAs that regulate thousands of protein-coding genes by binding to the 3’ untranslated region of the targeted messenger RNA (mRNA) [17]. Their ability to regulate (and serve as) tumor suppressors and oncogenes [17], their remarkable stability in formalin-fixed paraffin-embedded (FFPE) tissue [18] and biofluids [19], and their altered expression in PDACs compared to normal pancreas tissue [20] makes miRNAs excellent candidate biomarkers of early progression to pancreatic malignancy. Indeed, early studies of small numbers of miRNAs in tumor versus normal tissue supported a role for altered miRNA expression in PanINs [21] and IPMNs [22]. Since more than 1,000 miRNAs exist [23], several recent studies [24,25] have conducted focused investigations of genome-wide miRNA expression in IPMN tissue. Matthaei et al [24] profiled archived tissue and cyst fluid from patients with IPMNs and other pancreatic lesions and identified 9 miRNAs (miR-24, miR-18a, miR-30a-3p, miR-92a, miR-106b, miR-342-3p, miR-99b, miR-142-3p, miR-532-3p) that predicted cyst pathology with a sensitivity of 89% and a specificity of 100%, whereas Lubezky et al [25] highlighted four different miRNAs (miR-217, miR-21, miR-708, miR-155). While these studies [24,25] were underway, we also conducted a pilot investigation of genome-wide miRNA expression in IPMN tissue, with the goal of discovering miRNAs that may differentiate ‘high-risk’ IPMNs (defined by pathologically-confirmed invasive disease or high-grade dysplasia without associated invasion) that require resection from ‘low-risk’ IPMNs (defined by pathologically-confirmed low- or moderate-grade dysplasia) that can be monitored. Our study is novel in that we also aimed to explore a) associations between candidate miRNA expression and selected clinical and pathologic factors and b) genes and pathways regulated by the candidate miRNAs by integrating data from bioinformatic analyses and existing microarray analysis of miRNA gene targets.

## Materials and Methods

### Study population and biospecimens

A prospectively maintained clinical database was retrospectively reviewed to identify individuals who underwent pancreatic resection for an IPMN between 1999 and 2011 at Moffitt Cancer Center and Research Institute (Moffitt) and had provided written consent for tissue to be donated for research through several protocols approved by the Institutional Review Board (IRB) of the University of South Florida, including Total Cancer Care (http://moffitt.org/patient-services/total-cancer-care/research) [26]. IRB approval was also specifically granted for the research described herein (IRB# Pro4971). A pathologist with expertise in PDAC and IPMN pathology (DC) used hematoxylin and eosin (H&E) stained slides from selected blocks to histologically confirm the diagnosis and degree of dysplasia using World Health Organization guidelines [27], and consulted with another pancreatic pathologist (BC) as needed. The final diagnosis represented the most severe grade of dysplasia observed in the neoplastic epithelium of each resected lesion, and multiple representative areas of the corresponding grade were electronically marked on the H&Es. Examples of grades of IPMN dysplasia are shown in S1 Fig.

Laser capture microdissection (LCM) and RNA isolation

Under RNAse-free conditions, four 8-micron sections were cut from the FFPE block corresponding to each respective H&E. Sections were placed in a water bath, mounted on uncharged and uncoated glass slides, air-dried overnight, and transferred to Moffitt’s Analytic Microscopy Core for LCM. Sections were deparaffinized, hydrated, and stained using nuclease-free Histogene solution (Applied Biosystems (ABI), Austin, TX), dehydrated, and then air-dried before placement in an Autopix LCM instrument (Arcturus, Molecular Devices, Sunnyvale, CA). Locations of dysplasia were verified using the marked electronic images. Cells of interest were captured from each section using Macro LCM Caps (Arcturus CapSure, #09F10A). Caps of cells from each case were pooled and 50 uL of lysis buffer was added to stop RNA degradation. Qiagen’s miRNeasy FFPE Isolation Kit was used for total RNA isolation, which included isolation of small non-coding RNAs, according to the manufacturer’s procedures. RNA quantity and quality was assessed by Optical Density (OD) at 260 and 280 nm using a Nanodrop spectrophotometer. When RNA quantity was insufficient, additional tissue sections were processed. If RNA quality was poor, an ethanol precipitation was performed.

### High-throughput miRNA expression analysis

In our discovery phase we conducted genome-wide miRNA profiling using Taqman MicroRNA Arrays, also known as Taqman Low Density Array (TLDA) ‘Pool A’ Card version 3.0, due to their established use for miRNA expression analysis in FFPE tissue [28,29]. This 384-microfluidic array was designed to perform quantitative reverse transcriptase (qRT-PCR) reactions simultaneously (Applied Biosystems, Austin, TX, USA) on 378 mature miRNAs (and 6 endogenous controls) that tend to be functionally defined. Using 20 nanograms (ng) of total RNA as input, cDNA was synthesized with highly multiplexed Megaplex RT primers, pre-amplified with Megaplex PreAmp Primers, mixed with TaqMan Universal PCR Master Mix (Applied Biosystems), and loaded onto TLDA cards for expression analysis.

### Individual qRT-PCR validation of miRNA candidates

The most deregulated miRNAs were evaluated in an independent set of 21 IPMNs (13 high-risk and 8 low-risk) as part of a validation phase. Total RNA was isolated from microdissected cells, and singleplex qRT-PCR assays were performed using 10 ng total RNA per reaction using pre-designed Taqman MicroRNA Assays (Applied Biosystems, Foster City, CA). The expression level of the most stable and abundantly expressed endogenous control from the discovery phase (RNU44) was used for normalization. All assays were carried out in triplicate to ensure reproducibility. Positive and no-template control (nuclease free water) samples were used to evaluate reagent performance and contamination. PCR was run on the 7900HT instrument according to the manufacturer’s instructions. For each sample, the threshold cycle (Ct) was calculated by the ABI Sequence Detection software v2.3.

### Statistical Analyses

Descriptive statistics were determined using frequencies and percents for categorical variables and means and standard deviations (SD) for continuous variables. The distributions of covariates were compared across the low- versus high-risk IPMN groups using t-tests for continuous variables and Chi-squared or Fisher’s exact tests for categorical variables, as appropriate. Relative miRNA expression levels were calculated using a method similar to the comparative Ct (2^−∆∆CT^) method [30]. Briefly, ∆CT was calculated for each miRNA so that each miRNA was first normalized to the most stably expressed endogenous control, RNU44 (∆CT = CT -RNU44). Normalized CT values were further calculated as log_2_ [Max (∆CT)—∆CT]. The miRNA expression data for the discovery phase is available at: http://www.ncbi.nlm.nih.gov/geo/query/acc.cgi?acc=GSE63105.

Nonparametric tests (Wilcoxon rank sum tests) were performed to compare the normalized expression levels between groups for each miRNA. False discovery rates (FDR) adjusting for multiple comparisons were estimated using q-values [31]. Correlations between expression of the most deregulated miRNAs and selected clinical and pathological factors were explored using Pearson correlations for continuous variables and ANOVA and logistic regression for categorical variables. Multivariable regression analysis was conducted to identify miRNAs associated with high-risk IPMN status independent of selected variables. To assess the accuracy and clinical utility of candidate miRNAs in differentiating between high-risk and low-risk IPMNs, receiver operating characteristic (ROC) curves were constructed using ∆CT values, with pathological diagnosis as the gold standard. Logistic regression models predicting risk status were fit using values from the discovery dataset, and ROC curves were used to predict high- versus low-risk IPMN status in the validation dataset. A *P*-value <0.05 was used as the threshold for statistically significance in most analyses. All statistical analyses were performed using Matlab version 2009b and R version 2.13.1. To visualize miRNA expression patterns, we generated heatmaps and performed unsupervised, hierarchical clustering using Matlab.

### Bioinformatic Analyses

To gain insight into mechanisms responsible for miRNA-mediated progression to pancreatic malignancy, publicly-available tools were used to identify genes and pathways controlled by the candidate miRNAs. We determined experimentally-verified mRNA targets of the most deregulated miRNAs using the miRecords [32] and TarBase [33] databases and published literature. Using identified mRNAs as candidates, a pathway enrichment analysis was conducted using Gene Ontology’s MetaCore database (www.genego.com). Pathways related to PDAC and interaction hubs (genes with more than 5 interactions) were identified and overlapped with gene targets to narrow down the number of biologically important genes.

We also conducted additional analyses to explore novel *predicted* miRNA:mRNA pairs that may be involved in the in the histological progression of IPMNs. We used the miRWalk tool [34] because it hosts predicted miRNA-target interaction information for more than 2,000 miRNAs on the complete sequence of all known genes produced by both miRWalk and 8 established miRNA-target prediction programs (Diana microT v 3, miRanda August 2010, miRDB April 2009, PICTAR March 2007, PITA Aug 2008, RNA22 May 2008, RNAhybrid v 2.1, and Targetscan 5.1).

### Microarray gene expression analysis of IPMNs

Prior to the current miRNA-based investigation, under an IRB-approved protocol [26] frozen tumor tissue from patients treated at Moffitt was previously arrayed on Affymetrix HuRSTA-2a520709 GeneChips (Affymetrix, Santa Clara, CA) which contained ~60,000 probe sets representing ~25,037 unique genes (Affymetrix HuRSTA-2a520709, GEO: http://www.ncbi.nlm.nih.gov/geo/query/acc.cgi?acc=GPL10379). Of distinct solid tumors that were arrayed, 23 represented surgically-resected, pathologically-confirmed IPMNs (17 invasive and 6 non-invasive (1 low-grade, 1 moderate-grade, 4 high-grade)). For the 23 IPMNs that were arrayed, expression data for experimentally-validated and predicted gene targets of interest highlighted by bioinformatics analysis were normalized using Robust Multi-array Average and then extracted. Due to small cell counts, the high-risk group represented all invasive IPMNs and was compared to a low-risk group that included all non-invasive IPMNs. Gene expression data for the 23 IPMNs is available at: http://www.ncbi.nlm.nih.gov/geo/query/acc.cgi?acc=GSE63105. Nonparametric tests (rank sum tests) were used to compare expression between groups for each target gene. FDRs were estimated using q-values [31]. Of the 23 IPMN cases with microarray data, 8 had available tissue that was evaluated as part of the current miRNA discovery (n = 2; 1 low-grade, 1 high-grade) or validation phase (n = 6; 1 moderate-grade, 3 high-grade, 2 invasive), which enabled preliminarily exploration of miRNA:target mRNA expression relationships for paired samples using Pearson correlations.

## Results

### Study Population

Of 58 IPMN cases that were pathologically evaluated, 49 contributed tissue for the discovery (n = 28) or validation phase (n = 21). In the discovery phase, 19 cases were high-risk (all high-grade without invasion) and 9 were low-risk (all low-grade). In the validation phase, 13 cases were high-risk (11 high-grade without invasion, 2 invasive) and 8 were low-risk (4 low-grade and 4 moderate-grade). Clinical and pathologic characteristics of the 32 high-risk and 17 low-risk IPMN participants are shown in Table 1. Overall, characteristics were not significantly different between the high- and low-risk groups (Table 1). Age at diagnosis was slightly older in individuals with high-risk IPMNs (69.1 years) compared to those with low-risk IPMNs (65.1 years). The predominant tumor location for 59% of the high-risk IPMNs was the pancreatic head, whereas most (65%) low-risk IPMNs occurred in the pancreatic body or tail. On endoscopic ultrasound (EUS), signs of malignant potential were observed more frequently among high-risk (72%) compared to low-risk IPMNs (47%) (*P* = 0.09). Only 40% of high-risk IPMNs were observed to be > 3 cm on EUS. High-risk IPMNs were significantly more likely to involve the main pancreatic duct upon pathological review compared to low-risk IPMNs (*P* = 7× 10^−4^). The distribution of characteristics was similar among cases in each phase.

**Table 1:** Clinical and Pathologic Characteristics of Patients with IPMNs (N = 49).

**Variable**	**Low-risk^1^ IPMNs (n = 17)**	**High-risk^2^ IPMNs (n = 32)**	***P*-value^3^**
**Age at diagnosis, mean (SD)(yrs)**	65.1 (9.6)	69.1 (9.7)	0.18
**Gender**			
**Male**	11 (65)	18 (56)	0.57
**Female**	6 (35)	14 (44)	
**Race**			
**White, Non-Hispanic**	15 (88)	29 (91)	0.79
**Other**	2 (12)	3 (9)	
**Year of Surgery**			
**1999–2005**	1 (6)	5 (16)	0.32
**2006–2011**	16 (94)	27 (84)	
**Predominant tumor location**			
**Pancreatic Head**	6 (35)	19 (59)	0.11
**Pancreatic Body or Tail**	11 (65)	13 (41)	
**Signs of malignant potential ^4^on EUS**	8 (47)	23 (72)	0.09
**Size of largest cyst on EUS ^4^**			
**<3 cm.**	14 (82)	18 (60)	0.11
**≥3 cm.**	3 (18)	12 (40)	
**Size of largest cyst^4^, mean (SD) (cm)**	2.0 (1.2)	2.5 (1.3)	0.21
**Pancreatic duct involvement ^5^**			
**Main duct or mixed**	5 (29)	25 (78)	**7 × 10^−4^**
**Side branch duct**	9 (53)	4 (13)	
**Asymptomatic**			
**Yes**	3 (18)	4 (13)	0.62
**No**	14 (82)	28 (88)	
**Personal history of chronic pancreatitis**			
**Yes**	6 (35)	16 (50)	0.32
**No**	11 (65)	16 (50)	
**Family history of pancreatic cancer**			
**Yes**	1 (6)	2 (6)	0.97
**No**	15 (88)	29 (91)	
**Ever Smoker**			
**Yes**	11 (65)	20 (63)	0.88
**No**	6 (35)	12 (38)	

Data represent counts (percentages) unless otherwise indicated. Counts may not add up to the total due to missing values, and percentages may not equal 100 due to rounding.

^1^Low-risk IPMNs are represented by 12 low-grade and 5 moderate-grade IPMNs.

^2^High-risk IPMNs are represented by 30 high-grade and 2 invasive IPMNs.

^3^P-value for differences between low- and high-risk groups using chi-squared or Fisher’s exact tests and t-tests for categorical and continuous variables, respectively. Values in bold are statistically significant (P<0.05).

^4^Signs of malignant potential on endoscopic ultrasound (EUS) include main duct (MD) involvement, MD dilation (≥5 mm), mural nodules, septation, wall thickness, or cyst size ≥3 cm.

^5^Based on pathological review post-resection.

### Biospecimen quality

**Figure 1:**
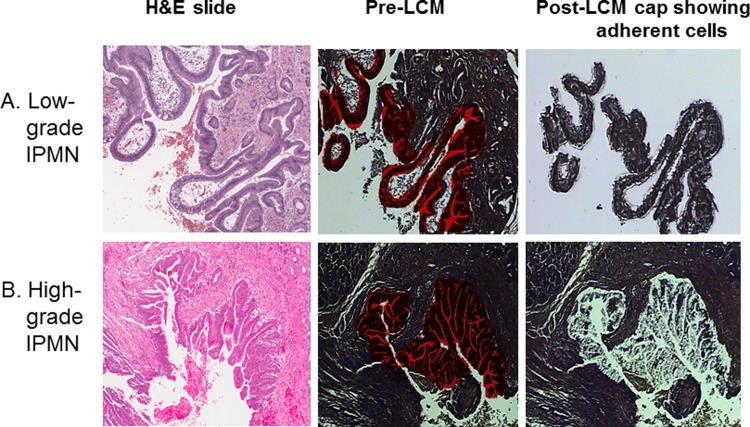
Laser capture microdissection (LCM) of epithelium from A) low- grade and B) high-grade IPMN tissue. Left Panel: Hematoxylin (H&E) stained slide (× 4). Middle Panel: H&E stained slide before LCM (× 4), with the red area representing cells of interest marked for capture. Right Panel: Cap showing adherent cells.

Surgically-resected tissue was pathologically evaluated for 58 unique IPMNs. Tissue was not profiled for 6 cases due to an inconclusive grade of dysplasia (n = 1), sparse regions of dysplasia (n = 2), or technical issues during LCM (n = 3). Representative examples of pre- and post- microdissection images of low- and high-grade IPMNs are shown in Fig. 1A and 1B, respectively. The average total number of cells captured per case was 8,498 (range: 780–65,956), and the average total RNA recovery was 139 ng (range: 48–591 ng). The quality of RNA was appropriate for most cases as evidenced by optical density 260/280 readings in the range of 1.8–2.0. Sub-optimal RNA quantity or quality was observed for 3 additional cases, leaving 49 cases with adequate tissue for miRNA expression analyses.

### miRNA expression analysis in the discovery and validation phase

**Figure 2:**
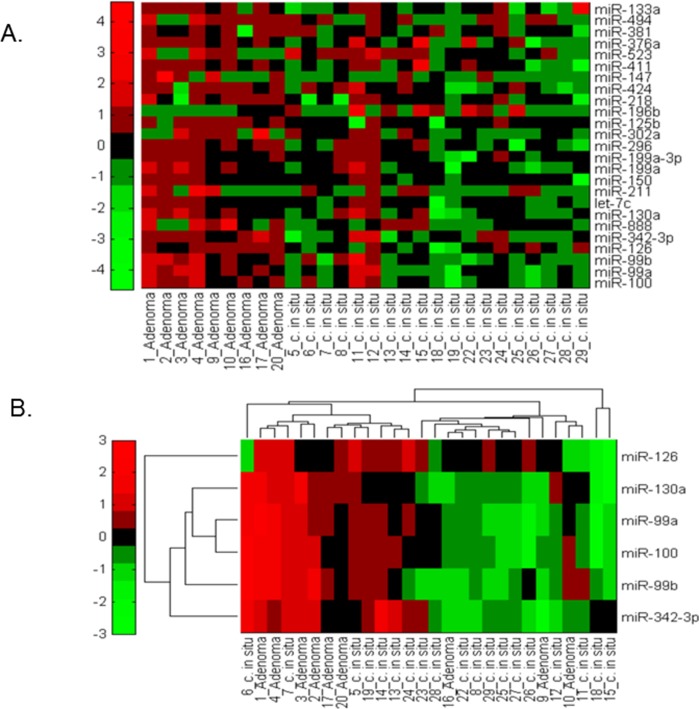
Heatmap and unsupervised hierarchical clustering of low-risk (adenoma) and high-risk (carcinoma-in-situ) IPMN samples according to the expression of the most differentially expressed miRNAs. A) The heatmap is supervised, and is ordered by the type of IPMN, and shows the expression for the 25 most deregulated miRNAs. B) Unsupervised hierarchical clustering for the 6 most differentially expressed miRNAs. Expression values for the miRNAs are represented in a matrix format, with columns representing samples and rows representing miRNAs. Low expression values are colored green, and high expression values are colored red. Colored bars indicate the range of normalized log2-based signals.

In the discovery phase, 236 of 378 miRNAs evaluated (62.4%) were detectable in at least half of the 28 samples evaluated and were included in subsequent analyses. This percentage is comparable to other studies [24]. Thirty-five miRNA probes were significantly deregulated in high-risk versus low-risk IPMNs (rank sum *P*<0.05, S1 Table). The top deregulated miRNAs separated most low-risk from high-risk IPMNs as shown in the heatmap (Fig. 2A), though outliers existed, consistent with other studies [25]. Several outliers may be explained by focal areas of dysplasia available for sampling and/or inter-sample heterogeneity.

Using a FDR of 10%, 13 of the 35 miRNAs (miR-100, miR-99b, miR-99a, miR-342-3p, miR-126, miR-888, miR-130a, let-7c, miR-150, miR-296, miR-199a, miR-199a-3p, and miR-302a) were significantly deregulated between the groups (S1 Table). Of these 13 miRNAs, we selected the top 6 (miR-100, miR-99b, miR-99a, miR-342-3p, miR-126, miR-130a) for further evaluation based on their statistical significance, evidence to support their biological role in pancreatic carcinogenesis, and the fact that they were detectable in all evaluated samples. Our analysis showed that expression levels of each of these 6 miRNAs were *down*-regulated in most high- versus low-risk IPMNs (Table 2). Unsupervised hierarchical clustering analysis also illustrates reduced expression for these 6 miRNAs in the high-risk compared to the low-risk group (Fig. 2B). Experimentally-validated gene targets of the 6 miRNAs are listed in Table 2, and include well-known oncogenes.

**Table 2:** Select candidate miRNAs differentially expressed in high- (N = 19) vs. low-risk (N = 9) IPMN tissue.

**miRNA**	**P-value^1^**	**Median Fold change^2^**	**Mean Fold change^2^**	**Experimentally validated gene target(s)^3^**
**miR-100**	1.6 × 10^−3^	5.9	4.9	*ATM, FGFR3, IGF1R, MMP13, mTOR, PLK1, RPTOR*
**miR-99b**	2.7 × 10^−3^	4.7	3.7	*RAVER2*
**miR-99a**	2.7 × 10^−3^	4.8	4.7	*AGO2, COX2, FGFR3, IGF1R, MEF2D, mTOR, RAVER2, RPTOR, SERPINE1, SKI, TRIB1*
**miR-342–3p**	3.7 × 10^−3^	4.8	3.3	*BMP7, DNMT1, GEMIN4*
**miR-126**	3.7 × 10^−3^	3.1	6.7	*ADAM9, CCNE2, CRH, CRK, CRKL, DNMT1, EGFL7, KRAS, HOXA9, IRS1, PGF, PIK3R2, PLK2, PTPN7, RGS3, SLC45A3, SOX2, SPRED1, TOM1, TWF2, VCAM1, VEGFA*
**miR-130a**	5.9 × 10^−3^	4.7	5.0	*APP, ATG2B, ATXN1, CSF1, DICER1, ESR1, HOXA10, HOXA5, KLF4, MAFB, MEOX2, PPARG, RUNX3, TAC1, TP53INP1*

^1^Wilcoxon rank-sum test.

^2^All fold-changes represent *decreased* expression in the high-risk group (all high-grade IPMNs) versus the low-risk group (all low-grade IPMNs).

^3^According to data in Tarbase (http://diana.cslab.ece.ntua.gr/tarbase/), miRecords (http://mirecords.biolead.org/), or other sources.

In the validation phase, the top 6 miRNAs were evaluated in 21 independent IPMNs (13 high-risk, 8 low-risk) (S2 Fig.). We observed the same trends in expression as in the discovery phase, with the expression of each miRNA *down*-regulated in high-risk versus low-risk IPMNs (S3A and S3B Fig.). Using a threshold of *P*<0.05, results reached marginal statistical significance, possibly due to the small sample size. Among the 6 miRNAs evaluated, miR-130a was most strongly associated with high-risk IPMN status (2.9 median fold-change between the high- and low-risk group, *P* = 0.065), followed by miR-99b (2.7 median fold-change, *P* = 0.103) and miR-100 and miR-342-3p (2.2 median fold-change, *P* = 0.119). The miRNA data analyzed in the validation phase can be found in the S1 Supporting Information.

Most clinical and pathological factors were not correlated with miRNA expression in the discovery phase (S2 Table). However, an association was observed between low miR-99b expression and main duct involvement (*P* = 0.021), a variable independently associated with high-risk IPMN status (*P* = 0.044). After including both miR-99b expression level and main duct involvement in multivariate regression models, low miR-99b expression was marginally associated with high-risk IPMN status (*P* = 0.051). Serum albumin levels were positively correlated with miR-99a (r = 0.52, *P* =0.004) and miR-100 expression (r = 0.49, *P* = 0.008). The expression level of several miRNAs (miR-99a, miR-99b, miR-100) was highly correlated (0.79<r<0.97), which may be expected since they are from the same miRNA family [23](S2 Table). No factors were associated with miRNA expression (*P*<0.05) in the validation phase.

**Figure 3:**
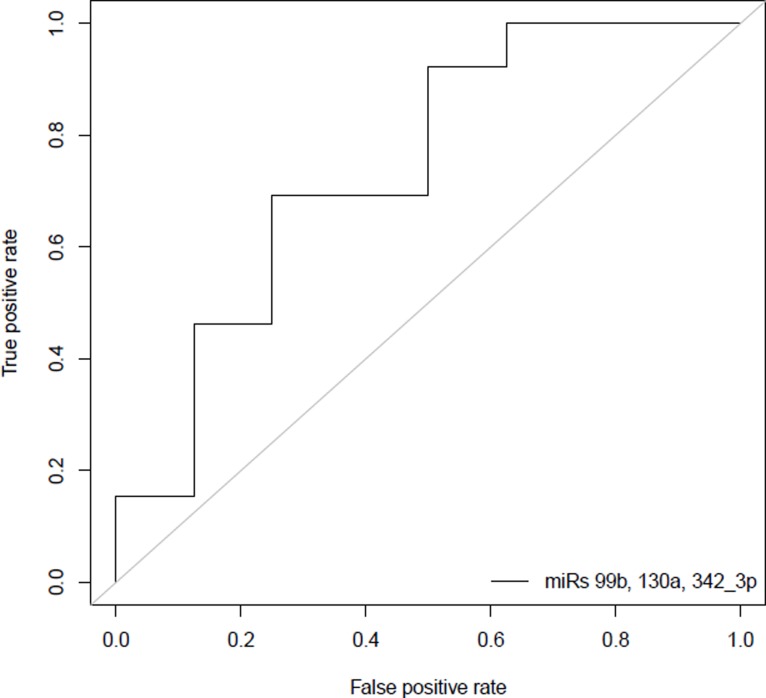
Receiver operating characteristic (ROC) curve analysis using miRNA expression to discriminate high-risk from low-risk IPMN samples. Using a logistic regression model built on data from the discovery dataset, a miRNA signature consisting of miR-99b, miR-130a, and mir-342-3p yielded an area underneath the curve (AUC) value of 0.74 (95% CI: 0.51–0.97) in differentiating between 13 high-risk and 8 low-risk IPMNs in the validation phase.

Receiver operating characteristic (ROC) curves were constructed based on the expression of miR-100, miR-99b, miR-99a, miR-342-3p, miR-126, and miR-130a. Most areas underneath the curve (AUC) values were comparable for the six individual miRNAs, with expression of miR-99a yielding the highest AUC value of 0.87 in classifying between high- and low-risk IPMNs in the discovery phase (S4 Fig.). When using a signature consisting of three miRNAs (miR-99b, miR-130a, miR-342-3p) from the discovery model to predict high-risk IPMN status in the validation set, the AUC was 0.74 (95% CI:0.51–0.97) (Fig. 3). A model combining expression of miR-99b, miR-130a, and miR-342-3p with presence of main duct involvement enabled slightly higher utility in discriminating between groups (AUC = 0.81).

### Follow-up with bioinformatic analyses and gene expression profiling

Gene ontology pathway enrichment network analysis of *experimentally-validated* miRNA gene targets listed in Table 2 revealed several important interaction hubs, including *ESR1, PPARG, VEGF-A, mTOR, IRS-1,* and *SOX2* (S5 Fig.). The top four most marked gene ontology maps that were identified contribute to tumor progression through interactions with histone deacetylase and calcium/calmodulin-dependent kinases, hypoxia inducible factor regulation, growth factor signaling, and cytoskeleton remodeling (Table 3). This analysis also confirmed that identified target genes were associated with pancreatic diseases (*P* = 2.2 × 10^−30^) and pancreatic neoplasms (*P* = 7.6 × 10^−29^).

**Table 3:** Gene ontology categories of biological pathways overrepresented by miRNA-mediated changes in target gene expression that may differentiate between high- and low-risk IPMNs.

**Pathway**	**P-value**	**False Discovery Rate (FDR)**
Developmental role of histone deacetylase (HDAC) and calcium/calmodulin-depdendent kinase (CaMK)	9.5 × 10^−8^	2.4 × 10^−5^
Transcription receptor-mediated hypoxia inducible factor (HIF) regulation	5.9 × 10^−7^	7.5 × 10^−5^
Developmental membrane-bound ESR1: interaction with growth factors signaling	1.3 × 10^−6^	1.0 × 10^−4^
Cytoskeleton remodeling with TGF and WNT	6.9 × 10^−6^	3.8 × 10^−4^
DNA damage with BRCA1 as a transcription regulator	7.5 × 10^−7^	3.8 × 10^−4^
Signal transduction-AKT signaling	3.3 × 10^−5^	8.9 × 10^−4^
Development: VEGF signaling and activation	3.3 × 10^−5^	8.9 × 10^−4^
Development: ligand-independent activation of ESR1 and ESR2	3.9 × 10^−5^	8.9 × 10^−4^
Role of alpha-6/beta-4 integrins in carcinoma progression	3.9 × 10^−5^	8.9 × 10^−4^

Gene expression was significantly up-regulated in 17 high-risk versus 6 low-risk IPMNs for *DNMT1, ATG2B and MEOX2,* and *IRS1* (*P*<0.10), which are experimentally-validated targets for miR-342-3p, miR-130a, and miR-126, respectively. A total of 297 unique genes represented by 458 probes were identified as *predicted* targets of the 6 miRNAs highlighted in our investigation by seven or more target prediction programs. When reviewing the gene expression data, there are 40 genes differentially expressed between high- and low-risk IPMNs (P<0.10), with some being down-regulated and some being up-regulated by the candidate miRNAs, and miR-130a in particular (S3 Table). In the small subset of IPMN cases with miRNA and mRNA expression data, positive correlations between miRNA and mRNA expression were evident in 2 miRNA-mRNA pairs, *miR-342-3p: DNMT1* (r = 0.81, *P* = 0.05) and *miR-126: IRS1* (r = 0.78, *P* =0.07). No statistically significant inverse correlations between miRNAs and their target genes were observed; this is contrary to the anti-correlated expression that is expected and may be attributed to the small number of IPMN cases with both data types.

## Discussion

We conducted a genome-wide miRNA expression analysis exclusively in ‘high-risk’ and ‘low-risk’ IPMNs that was followed by both a validation and a follow-up phase, and discovered biologically-meaningful miRNAs that may help distinguish between these groups. Six miRNAs (miR-100, miR-99b, miR-99a, miR-342-3p, miR-126, and miR-130a) were under-expressed in high-risk compared to low-risk IPMNs in our discovery and validation phase, suggesting that low or reduced levels of these miRNAs (and possibly increased levels of target genes they regulate) may be associated with progression to invasion. Moreover, ROC analysis suggested a combination of miRNAs may help to classify IPMNs based on histologic severity (Fig. 3).

Consistent with our findings, data support a role for the identified miRNAs as tumor suppressors and regulators of oncogenes that contribute to cell proliferation and invasion in pancreatic and other malignancies (Table 2)[35–42]. For example, an experimentally-validated target of miR-100 is polo-like kinase 1 (*PLK1*) [38,39], a regulator of proliferative activity over-expressed in early PDAC [41] that represents a novel target for chemoprevention and therapeutic strategies [41,43]. Also noteworthy, miR-342-3p can inhibit cancer cell proliferation and invasion by targeting DNA methyltransferase 1 (*DNMT1*), a gene that maintains DNA methylation [40]. *DNMT1* mRNA expression has been correlated with PDAC progression; those with higher *DNMT1* tissue expression had poorer survival than those with lower expression [42]. Loss of another candidate, miR-126, has been associated with PDAC progression by targeting oncogenes such as *KRAS* [36,37] and insulin receptor substrate-1 (*IRS-1*), a mediator of phosphoinositide 3-kinase (PI3K) activation in quiescent PDAC cells [44]. Pathway enrichment network analysis underscored that these and other miRNA-mediated mechanisms may explain how IPMNs progress to invasive disease (Table 3).

Even though miRNAs are generally believed to regulate expression at the protein level [17], we postulated that measuring mRNA expression may be useful for determining differences in transcriptional machinery between a pre-malignant and a malignant state. Consistent with our predictions, mRNA targets of miR-130a (*ATG2B* and *MEOX2),* miR-342-3p *(DNMT1),* and miR-126 (*IRS-1*) were up-regulated in high-risk versus low-risk IPMNs. Other miRNA targets may not have demonstrated noticeable mRNA level changes since mRNA and protein abundance may not be strongly correlated [45]. We also explored correlations between miRNA and mRNA expression in the reduced set of IPMNs with both data types. Although positive correlations were observed in 2 miRNA-mRNA pairs (*miR-342-3p: DNMT1* (r = 0.81, *P*= 0.05) and *miR-126: IRS1* (r = 0.78, *P* = 0.07)), no statistically significant inverse miRNA:target gene correlations were observed. Indeed, previous studies observed more positively-correlated than negatively-correlated interactions [46], supporting a positive regulatory role of miRNAs [47]. Due to the small sample of IPMNs evaluated and the fact that paired miRNA:mRNA expression data was not available for all evaluated samples, caution should be taken when interpreting these mRNA-based findings. Tissue microarrays are being constructed on a larger series of IPMNs so that protein expression can be evaluated.

When the current investigation was underway, studies from the United States [24], Korea [48], Israel [25], and Europe [49] also focused on examining miRNA expression in IPMN tissue, but only Matthaei et al [24] and Lubezky et al [25] conducted genome-wide miRNA profiling (S4 Table). Matthaei and colleagues [24] evaluated miRNA expression in 22 IPMN tissue and 7 pancreatic cyst fluid (CF) samples, and performed validation using 23 additional IPMN FFPE samples and CF samples. miR-342-3p and miR-99b were among the miRNAs that differentiated between high-and low-risk groups using FFPE tissue (and CF) [24], demonstrating consistency of findings across our two studies and providing a form of external validation. Additionally, in a recent study by Lee et al [50] that aimed to identify miRNAs that distinguish between mucinous (IPMNs and MCNs) and non-mucinous pancreatic cysts (serous cystadenomas), several of the miRNA families identified by our study and/or Matthaei et al. [24] helped differentiate between mucinous and non-mucinous cysts (miR-99) and within mucinous cyst types (miR-130). No overlap existed between the main findings observed by Lubezky et al [25] and the current study. Since study population characteristics, sample preparation procedures, platforms (qRT-PCR versus microarray), normalization approaches, and other factors differed between studies, it is not surprising that different miRNAs were characteristic of high-risk IPMN status (S3 Table).

Although the sample sizes of our discovery and validation phases were relatively modest, they were comparable or even larger than the aforementioned studies with regard to the number of high-grade cases without associated invasion that were evaluated. This is important clinically because it would be opportune to reliably detect high-grade dysplasia so intervention could occur *prior* to invasion. Also of clinical importance, low miR-99b expression was associated with main duct involvement, a marker of histologic progression that was *not* observed pre-operatively by imaging for six high-grade cases in our study. We also observed positive correlations between serum albumin levels and expression of miR-99a and miR-100, and showed higher serum albumin levels in cases with low-risk IPMNs. This is in line with evidence that high serum albumin is correlated with better survival in PDAC patients [51], inferring it may be helpful to monitor serum albumin in patients with IPMNs since lower levels may be suggestive of a possible obstruction caused by a high-risk IPMN involving the main pancreatic duct. Larger prospective studies are needed to validate these observations.

Thus far, we are aware of only 5 study patients (10.2%) that went on to develop metastatic PDAC within a four year follow-up period; 1 was initially diagnosed with moderate-grade dysplasia; two had high-grade dysplasia at diagnosis; and 2 were initially diagnosed with localized invasive disease. All had main duct involvement at the initial diagnosis. Given that nearly 90% of study patients did not develop invasion or metastasis in the years post resection, our single institutional experience reiterates that the detection and management of high-risk IPMNs can offer a unique opportunity to prevent malignancy and improve outcomes.

Strengths of our pilot study include the well-annotated tissue and sound methodologic approach which capitalized upon confirmatory pathological review, standardized procedures for microdissection and RNA isolation of multiple representative regions per case, an established platform for miRNA profiling, and our follow-up using bioinformatic tools and available microarray data. Internal validity is evidenced by high correlations between expression levels of miRNAs from the same family. Among the limitations, we only analyzed 378 of the estimated 1,800 human miRNAs identified to date. Although we focused on this panel of well-defined miRNAs because of their higher detection rate and lower background signals, novel miRNAs not evaluated here may help distinguish between high- and low-risk IPMNs. It is noteworthy, however, that all but one of the candidate miRNAs highlighted in recent studies of IPMNs [24,25] were evaluated in the current study. Another possible limitation of the current study is that samples from the discovery phase that had been profiled using the Taqman MicroRNA Arrays were not evaluated in the validation phase using single qRT-PCR assays. Despite this limitation, it is meaningful that the same trends in expression were observed in the validation phase using an independent series of IPMN tissues and a different platform.

Taken together, the new miRNAs identified here (miR-99a, miR-100, miR-126, miR-130a) and those also highlighted by others (miR-99b, −342-3p) warrant evaluation in a well-powered multicenter prospective cohort of individuals presenting with pancreatic cysts. Moreover, given that miRNAs are released from tissues into circulation in a stable form protected from endogenous RNAse activity [52] and preliminary data support the clinical utility of miRNAs circulating in pancreatic juice or aspirate [22,53], cyst fluid [24], and serum [54], there is great potential for a minimally-invasive miRNA-based assay to be used to classify newly-diagnosed pancreatic cysts based on their malignant potential. Such an assay should be evaluated in conjunction with clinical and pathologic factors and emerging markers [55,56] to increase sensitivity and specificity. Furthermore, functional evaluation of the emerging miRNAs and their target genes may aid in understanding the molecular underpinnings of progression to pancreatic malignancy so that novel prevention and early detection strategies can be developed. In conclusion, a miRNA signature has the potential to serve as a promising diagnostic adjunct for directing management of IPMNs toward watchful waiting or resection.

## SUPPORTING INFORMATION

S1 Table The top 35 most differentially expressed miRNAs between high-risk (N = 19) and low-risk IPMNs (N = 9). (PDF)

S2 Table Correlations between candidate miRNA expression level and selected continuous clinical and pathologic characteristics. (PDF)

S3 Table Candidate MiRNAs and their Predicted Target Genes and their Expression in Invasive (n = 17) versus Non-invasive IPMNs (n = 6), sorted by statistical significance. (XLS)

S4 Table Studies of tissue-based miRNA expression in surgically-resected IPMNs. (PDF)

S1 Fig Representative histologic images of IPMNs with A) low-grade, B) moderate-grade, and C) high-grade dysplasia and D) invasive carcinoma. The region enclosed by the black line represents the area isolated by LCM. Reference bar = 50 micrometers (μm).
(PDF)

S2 Fig Schema illustrating the three study phases. In the discovery phase, genome-wide miRNA expression profiling of formalin-fixed paraffin-embedded (FFPE) tissue from 28 IPMNs was conducted. This was followed by a validation phase in which the six most degregulated miRNAs from the discovery phase (miR-100, miR −99b, miR-99a, miR-342-3p, miR-126, and miR-130a) were evaluated in an independent set of 21 IPMNs, while accounting for pertinent clinical and pathologic variables. In the final phase, a two-pronged approach was used to follow up findings: a) bioinformatic analyses were conducted to identify genes and pathways regulated by the candidate miRNAs and b) analysis was performed for candidate genes believed to be regulated by the identified miRNAs using existing microarray data for 23 IPMNs.
(PDF)

S3 Fig Box plots of candidate miRNA expression in IPMN tissue by real-time PCR. A) Discovery phase B) Validation phase. On each boxplot, the central mark is the median, and the edges of the box are the 25th and 75th percentiles. The whiskers extend to the most extreme data points within 1.5 of the interquartile range above the 75^th^ or below the 25^th^ percentiles. Data points beyond the whiskers, displayed using “o”, are potential outliers.
(PDF)

S4 Fig Receiver operating characteristic (ROC) curve analysis using miRNA expression to discriminate high-risk from low-risk IPMNs in the A) discovery and B) validation phase. (PDF)

S5 Fig Network of genes regulated by candidate miRNAs (miR-100, miR-99a, miR-99b, miR-342-3p, miR-126, and miR-130a) that were found to be differentially expressed between high- and low-risk IPMNs. (PDF)

S1 Supporting Information Raw Ct Counts from the Validation Phase. (XLS)
