# Supplementary material for: A Genome-Wide Investigation of MicroRNA Expression Identifies Biologically-Meaningful MicroRNAs That Distinguish between High-Risk and Low-Risk Intraductal Papillary Mucinous Neoplasms of the Pancreas
Source: PLoS One. 2015 Jan 21;10(1):e0116869. doi: 10.1371/journal.pone.0116869 (PMC4301643; doi:10.1371/journal.pone.0116869)
Supplement: S1 Table — (PDF) [file pone.0116869.s001.pdf]

Table S1. The top 35 most differentially expressed miRNAs between high-risk (N=19) and low-risk IPMNs (N=9)

miRNA probe	mean fold-change	median fold-change	P-value rank-sum test	False discovery rate	N[a] High-risk	N[a] Low-risk
hsa-miR-100-000437	4.90	5.86	0.0016	0.0929	19	9
hsa-miR-99a-000435	4.66	4.77	0.0027	0.0929	19	9
hsa-miR-99b-000436	3.75	4.74	0.0027	0.0929	19	9
hsa-miR-126-002228	6.73	3.09	0.0037	0.0929	19	9
hsa-miR-342-3p-002260	3.34	4.85	0.0037	0.0929	19	9
hsa-miR-888-002212	69.52	63.47	0.0057	0.0929	14	6
hsa-miR-130a-000454	5.01	4.72	0.0059	0.0929	19	9
hsa-let-7c-000379	3.59	2.66	0.0059	0.0929	18	9
hsa-miR-150-000473	3.49	2.78	0.0081	0.0929	18	9
hsa-miR-199a-000498	4.42	3.14	0.0081	0.0929	18	9
hsa-miR-199a-3p-002304	3.77	4.78	0.0081	0.0929	18	9
hsa-miR-296-000527	3.69	5.29	0.0081	0.0929	18	9
hsa-miR-302a-000529	36.30	6.42	0.0094	0.0995	12	5
hsa-miR-125b-000449	4.59	5.14	0.0113	0.1114	17	9
hsa-miR-218-000521	6.84	7.56	0.0131	0.1208	16	8
hsa-miR-424-000604	3.72	2.51	0.0156	0.1343	16	8
hsa-miR-411-001610	3.70	4.31	0.0168	0.1361	18	9
hsa-miR-523-002386	5.09	3.15	0.0196	0.1503	15	4
hsa-miR-376a-000565	3.41	6.76	0.0208	0.1509	19	9
hsa-miR-381-000571	4.35	6.96	0.0240	0.1535	19	8
hsa-miR-494-002365	3.57	3.36	0.0268	0.1535	11	7
hsa-miR-133a-002246	4.66	8.79	0.0269	0.1535	19	9
hsa-miR-139-5p-002289	3.37	4.63	0.0269	0.1535	19	9
hsa-miR-149-002255	8.29	12.69	0.0276	0.1535	11	9
hsa-miR-146b-3p-002361	11.48	22.42	0.0280	0.1535	10	5
hsa-miR-193a-5p-002281	2.25	1.85	0.0289	0.1535	17	8
hsa-miR-410-001274	3.12	3.98	0.0311	0.1589	17	9
hsa-miR-214-002306	3.96	5.68	0.0338	0.1637	16	9
hsa-miR-152-000475	2.15	3.12	0.0344	0.1637	19	9
hsa-miR-142-3p-000464	4.25	3.31	0.0368	0.1693	18	7
hsa-miR-30c-000419	2.73	3.27	0.0388	0.1728	19	9
mmu-miR-153-001191	0.09	0.05	0.0415	0.1789	12	6
hsa-miR-502-001109	4.63	7.01	0.0490	0.1936	15	8
hsa-miR-138-002284	3.70	3.96	0.0491	0.1936	19	9
hsa-miR-204-000508	6.44	9.55	0.0491	0.1936	19	9

[a] Number of IPMNs in which the miRNA was detectable.
