# Supplementary material for: A Genome-Wide Investigation of MicroRNA Expression Identifies Biologically-Meaningful MicroRNAs That Distinguish between High-Risk and Low-Risk Intraductal Papillary Mucinous Neoplasms of the Pancreas
Source: PLoS One. 2015 Jan 21;10(1):e0116869. doi: 10.1371/journal.pone.0116869 (PMC4301643; doi:10.1371/journal.pone.0116869)
Supplement: S2 Table — (PDF) [file pone.0116869.s002.pdf]

**Table S2. Correlations between candidate miRNA expression level and selected continuous clinical and pathologic characteristics.**

| | | miR_100 | | miR_99b | | miR_99a | | miR_342_3p | | miR_126 | | miR_130a | |
|---|---|---|---|---|---|---|---|---|---|---|---|---|---|
| | N | r | *P* | r | *P* | r | *P* | r | *P* | r | *P* | r | *P* |
| Age at diagnosis (years) | 28 | -0.14 | 0.47 | -0.02 | 0.93 | -0.16 | 0.42 | 0.19 | 0.34 | -0.25 | 0.2 | -0.18 | 0.37 |
| Size of the Largest Cyst (cm) | 28 | -0.1 | 0.63 | -0.15 | 0.45 | -0.21 | 0.28 | -0.24 | 0.21 | -0.2 | 0.32 | -0.19 | 0.34 |
| Size of the Main Pancreatic Duct (mm) | 15 | -0.48 | 0.07 | -0.49 | 0.07 | -0.33 | 0.23 | -0.17 | 0.55 | -0.13 | 0.65 | -0.49 | 0.07 |
| Fluid CEA levels (ng/uL) | 16 | -0.24 | 0.36 | -0.16 | 0.56 | -0.13 | 0.62 | -0.08 | 0.77 | -0.42 | 0.11 | -0.33 | 0.22 |
| Serum glucose levels (mg/dL) | 27 | -0.17 | 0.4 | -0.19 | 0.34 | -0.18 | 0.38 | -0.29 | 0.14 | 0.19 | 0.33 | -0.05 | 0.79 |
| Serum amylase level (u/L) | 10 | 0.4 | 0.25 | 0.24 | 0.5 | 0.214 | 0.55 | 0.44 | 0.2 | 0.54 | 0.11 | -0.17 | 0.65 |
| Serum CA19-9 level (U/ml) | 21 | 0.04 | 0.86 | 0.18 | 0.44 | -0.16 | 0.49 | -0.08 | 0.74 | -0.02 | 0.95 | 0.026 | 0.91 |
| Serum CEA level (ng/mL) | 7 | 0.32 | 0.49 | 0.02 | 0.96 | 0.3 | 0.51 | 0.31 | 0.5 | 0.46 | 0.3 | 0.468 | 0.29 |
| Serum albumin level (g/dL) | 28 | **0.49** | **0.01** | 0.31 | 0.11 | **0.524** | **0** | 0.37 | 0.05 | 0.09 | 0.67 | 0.253 | 0.19 |
| Serum bilirubin level (mg/dL) | 28 | 0.1 | 0.62 | 0.09 | 0.66 | 0.014 | 0.95 | 0.1 | 0.61 | 0.09 | 0.66 | -0 | 0.99 |
| Serum alkaline phosphatase level (u/L) | 28 | 0.17 | 0.39 | 0.1 | 0.63 | 0.08 | 0.69 | 0.14 | 0.49 | 0.1 | 0.61 | 0.081 | 0.68 |
| Body mass index (BMI) ($kg/m^2$) | 27 | -0.11 | 0.6 | -0.15 | 0.45 | -0.1 | 0.64 | 0.01 | 0.96 | 0.16 | 0.43 | 0.044 | 0.83 |
| Pack Years Smoked | 18 | **0.5** | **0.03** | **0.48** | **0.05** | 0.434 | 0.07 | 0.38 | 0.12 | 0.03 | 0.91 | 0.197 | 0.43 |
| miR-100 | 28 | 1 | NA | **0.82** | **0** | **0.969** | **0** | **0.7** | **0** | **0.43** | **0.02** | **0.864** | **0** |
| miR-99b | 28 | **0.82** | **0** | 1 | NA | **0.795** | **0** | **0.72** | **0** | 0.29 | 0.13 | **0.82** | **0** |
| miR-99a | 28 | **0.97** | **0** | **0.8** | **0** | 1 | NA | **0.72** | **0** | **0.44** | **0.02** | **0.858** | **0** |
| miR-342_3p | 28 | **0.7** | **0** | **0.72** | **0** | **0.715** | **0** | 1 | NA | 0.22 | 0.25 | **0.593** | **0** |
| miR-126 | 28 | **0.43** | **0.02** | 0.29 | 0.13 | **0.443** | **0.02** | 0.22 | 0.25 | 1 | NA | **0.569** | **0** |
| miR-130a | 28 | **0.86** | **0** | **0.82** | **0** | **0.858** | **0** | **0.59** | **0** | **0.57** | **0** | 1 | NA |

r = Pearson correlation
