# Supplementary material for: A Genome-Wide Investigation of MicroRNA Expression Identifies Biologically-Meaningful MicroRNAs That Distinguish between High-Risk and Low-Risk Intraductal Papillary Mucinous Neoplasms of the Pancreas
Source: PLoS One. 2015 Jan 21;10(1):e0116869. doi: 10.1371/journal.pone.0116869 (PMC4301643; doi:10.1371/journal.pone.0116869)
Supplement: S4 Table — (PDF) [file pone.0116869.s004.pdf]

Table S4. Studies of tissue-based miRNA expression in surgically-resected IPMNs.

	Habbe [20]	Matthaei [28]	Park [43]	Lubezky [29]	Caponi [44]	Permuth-Wey
Discovery phase						
**N IPMNs**[a]	**15** (non-inv)	**22** (10 LG, 12 HG[b])	**2** (1 LG, 1 HG)	**30** (10 LG, 5 MG, 5 HG, 10 inv)	**81** (16 non-inv, 65 inv)	**28** (9 LG,19 HG)
**Platform**	Taqman Singleplex qRT-PCR	Taqman MiRNA Array/ qRT- PCR	cDNA-mediated ligation	Gene Chip miRNA Array	Taqman Singleplex qRT-PCR	Taqman MiRNA Array/ qRT-PCR
**N miRNAs evaluated (normalization method)**	12 [c] (RNU6B[e])	750 ('diffpairs')	NA	850 (Robust multi-chip array algorithm)	3 [d] (RNU6[e])	378 (RNU44[e])
Replication phase[f]						
**N IPMNs**	**64** (13 LG, 31 MG, 20 HG)	**23** (3 LG, 9 HG, 11 inv)	**20** (NA)	**18** (9 LG, 9 inv)	**None**	**21** (4 LG, 4 MG, 11 HG, 2 inv)
**Platform (N miRNAs evaluated)**	LNA-ISH (2)	qRT-PCR (26[f])	qRT-PCR (NA)	qRT-PCR (4)	NA	qRT-PCR (6)
**Most deregulated miRNAs in high- versus low-risk IPMNs** [g]	**miR-21, -155**	miR-24,-18a, -30a-3p, -92a, -106b,**-342-3p** **-99b**, -142-3p, -532-3p	miR-552, -25, -182, -1300, -183 -196a, -30c	miR-217**, -21**, -708, **-155**	**miR-21,-155**	miR-100, -**99b**, -99a, **-342-3p**, -126, -130a

Abbreviations: IPMN=intraductal papillary mucinous neoplasm; inv=invasive; LG=low-grade; MG= moderate-grade; HG=high-grade; LNA-ISH=locked nucleic acid-*in situ* hybridization; NA=not available

[a] Some studies also evaluated normal pancreas tissue, PDAC tissue, and/or biofluids (pancreatic cyst fluid or pancreatic juice).

[b] 7 of the 12 HG IPMNs had associated invasive disease.

[c] The 12 evaluated miRNAs include: miR-15a, -16, 17-5p, -21, -100, -107, -155, -181a, -181c, -210, -221, -223.

[d] The 3 miRNAs that were evaluated include: miR-21,-155, -101.

[e] Endogenous control

[f] Represents the number of independent IPMNs in addition to those profiled in the discovery phase.

[g] miRNAs appearing in bold font were highlighted in more than one investigation. The 9 miRNAs highlighted by Matthaei et al are represented in their final predictive model which was also based on cyst fluid analysis.
