# Supplementary material for: A Genome-Wide Investigation of MicroRNA Expression Identifies Biologically-Meaningful MicroRNAs That Distinguish between High-Risk and Low-Risk Intraductal Papillary Mucinous Neoplasms of the Pancreas
Source: PLoS One. 2015 Jan 21;10(1):e0116869. doi: 10.1371/journal.pone.0116869 (PMC4301643; doi:10.1371/journal.pone.0116869)
Supplement: S1 Fig — The region enclosed by the black line represents the area isolated by LCM. Reference bar = 50 micrometers (μm). (PDF) [file pone.0116869.s005.pdf]

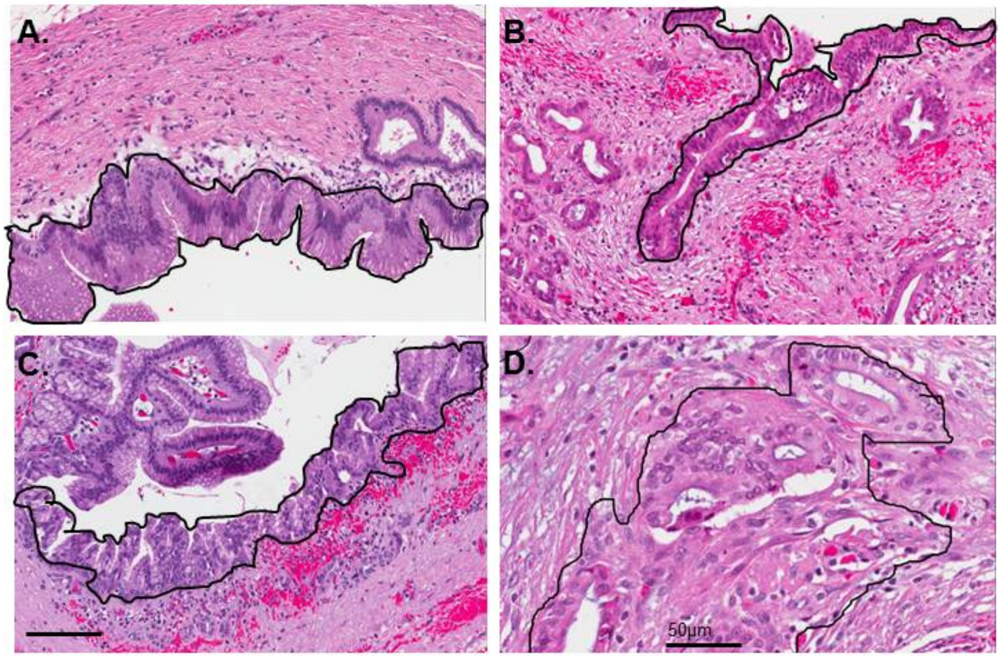

Figure S1. Representative histologic images of IPMNs with A) low-grade, B) moderate-grade, and C) high-grade dysplasia and D) invasive carcinoma. The region enclosed by the black line represents the area isolated by LCM. Reference bar= 50 micrometers (µm).
