# Supplementary material for: A Genome-Wide Investigation of MicroRNA Expression Identifies Biologically-Meaningful MicroRNAs That Distinguish between High-Risk and Low-Risk Intraductal Papillary Mucinous Neoplasms of the Pancreas
Source: PLoS One. 2015 Jan 21;10(1):e0116869. doi: 10.1371/journal.pone.0116869 (PMC4301643; doi:10.1371/journal.pone.0116869)
Supplement: S2 Fig — In the discovery phase, genome-wide miRNA expression profiling of formalin-fixed paraffin-embedded (FFPE) tissue from 28 IPMNs was conducted. This was followed by a validation phase in which the six most degregulated miRNAs from the discovery phase (miR-100, miR −99b, miR-99a, miR-342-3p, miR-126, and miR-130a) were evaluated in an independent set of 21 IPMNs, while accounting for pertinent clinical and pathologic variables. In the final phase, a two-pronged approach was used to follow up findings: a) bioinformatic analyses were conducted to identify genes and pathways regulated by the candidate miRNAs and b) analysis was performed for candidate genes believed to be regulated by the identified miRNAs using existing microarray data for 23 IPMNs. (PDF) [file pone.0116869.s006.pdf]

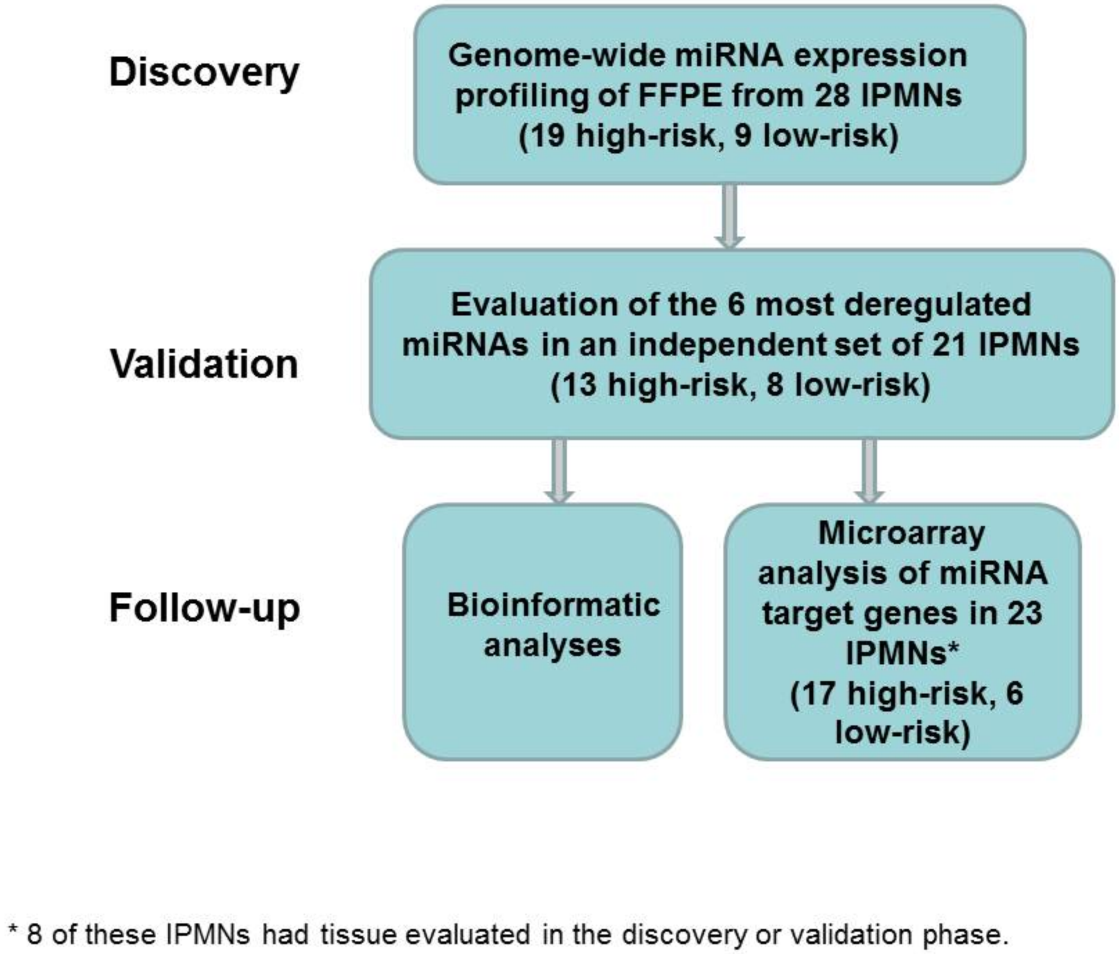

Figure S2. Schema illustrating the three study phases. In the discovery phase, genome-wide miRNA expression profiling of formalin-fixed paraffin-embedded (FFPE) tissue from 28 IPMNs was conducted. This was followed by a validation phase in which the six most degregulated miRNAs from the discovery phase (miR-100, miR -99b, miR-99a, miR-342-3p, miR-126, and miR-130a) were evaluated in an independent set of 21 IPMNs, while accounting for pertinent clinical and pathologic variables. In the final phase, a two-pronged approach was used to follow up findings: a) bioinformatic analyses were conducted to identify genes and pathways regulated by the candidate miRNAs and b) analysis was performed for candidate genes believed to be regulated by the identified miRNAs using existing microarray data for 23 IPMNs.
