# Supplementary material for: A Genome-Wide Investigation of MicroRNA Expression Identifies Biologically-Meaningful MicroRNAs That Distinguish between High-Risk and Low-Risk Intraductal Papillary Mucinous Neoplasms of the Pancreas
Source: PLoS One. 2015 Jan 21;10(1):e0116869. doi: 10.1371/journal.pone.0116869 (PMC4301643; doi:10.1371/journal.pone.0116869)
Supplement: S3 Fig — A) Discovery phase B) Validation phase. On each boxplot, the central mark is the median, and the edges of the box are the 25th and 75th percentiles. The whiskers extend to the most extreme data points within 1.5 of the interquartile range above the 75th or below the 25th percentiles. Data points beyond the whiskers, displayed using “o”, are potential outliers. (PDF) [file pone.0116869.s007.pdf]

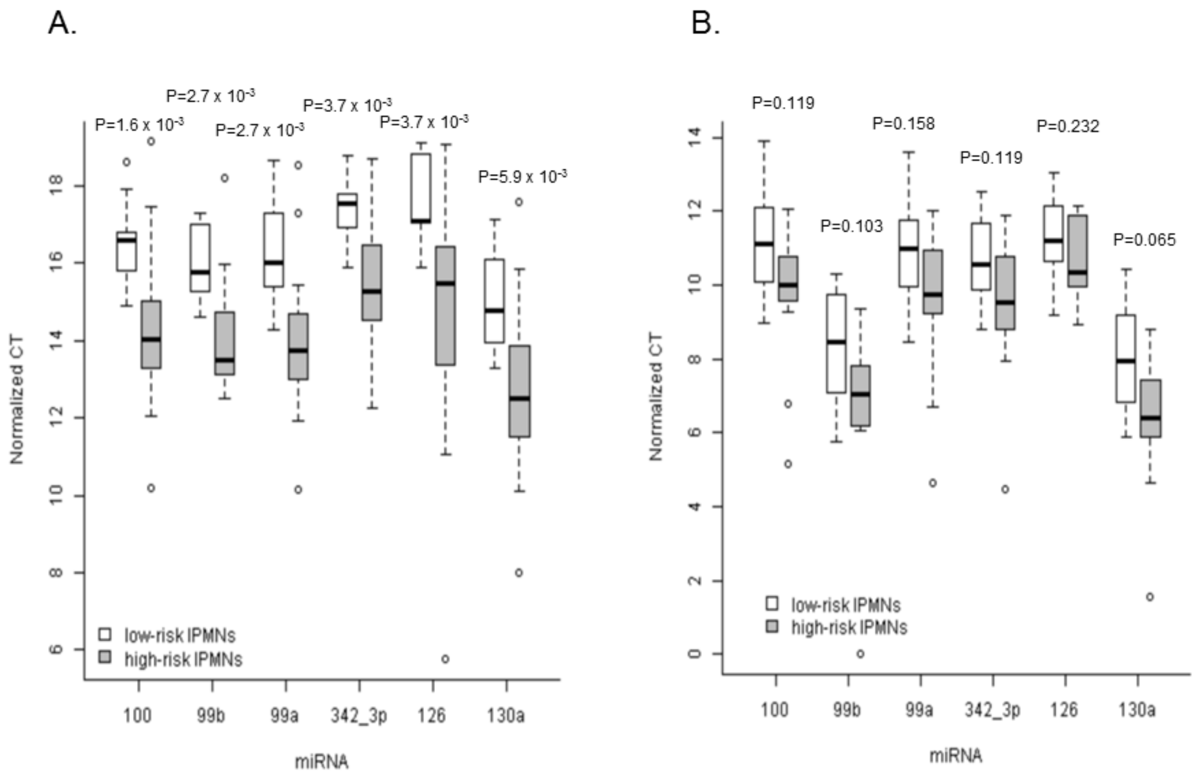

Figure S3. Box plots of candidate miRNA expression in IPMN tissue by real-time PCR. A) Discovery phase B) Validation phase. On each boxplot, the central mark is the median, and the edges of the box are the 25th and 75th percentiles. The whiskers extend to the most extreme data points within 1.5 of the interquartile range above the 75th or below the 25th percentiles. Data points beyond the whiskers, displayed using “o”, are potential outliers.
