# Supplementary material for: A Genome-Wide Investigation of MicroRNA Expression Identifies Biologically-Meaningful MicroRNAs That Distinguish between High-Risk and Low-Risk Intraductal Papillary Mucinous Neoplasms of the Pancreas
Source: PLoS One. 2015 Jan 21;10(1):e0116869. doi: 10.1371/journal.pone.0116869 (PMC4301643; doi:10.1371/journal.pone.0116869)
Supplement: S4 Fig — (PDF) [file pone.0116869.s008.pdf]

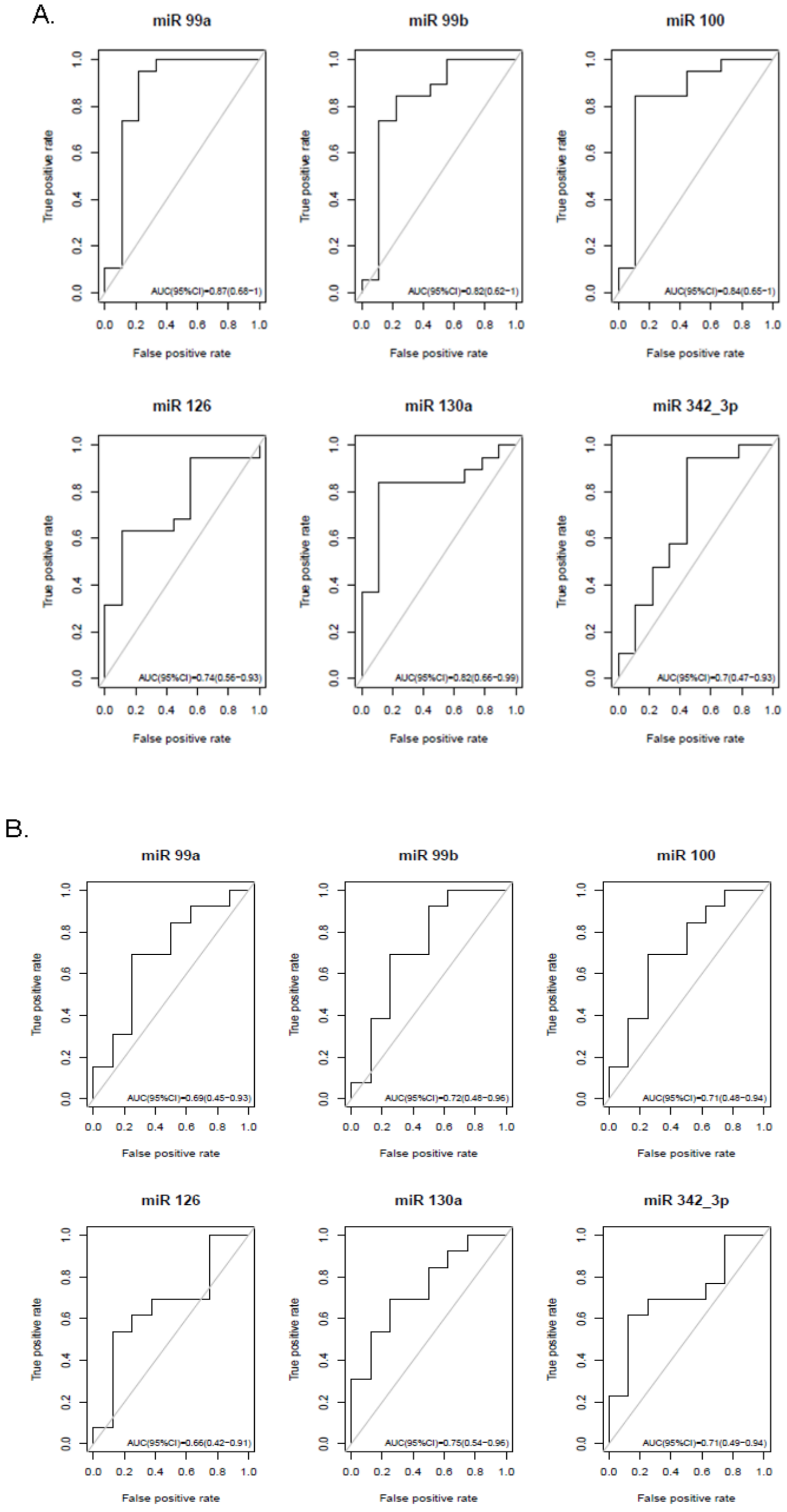


**Figure S4. Receiver operating characteristic (ROC) curve analysis using miRNA expression to discriminate high-risk from low-risk IPMNs in the A) discovery and B) validation phase.**
