# Supplementary material for: A Genome-Wide Investigation of MicroRNA Expression Identifies Biologically-Meaningful MicroRNAs That Distinguish between High-Risk and Low-Risk Intraductal Papillary Mucinous Neoplasms of the Pancreas
Source: PLoS One. 2015 Jan 21;10(1):e0116869. doi: 10.1371/journal.pone.0116869 (PMC4301643; doi:10.1371/journal.pone.0116869)
Supplement: S5 Fig — (PDF) [file pone.0116869.s009.pdf]

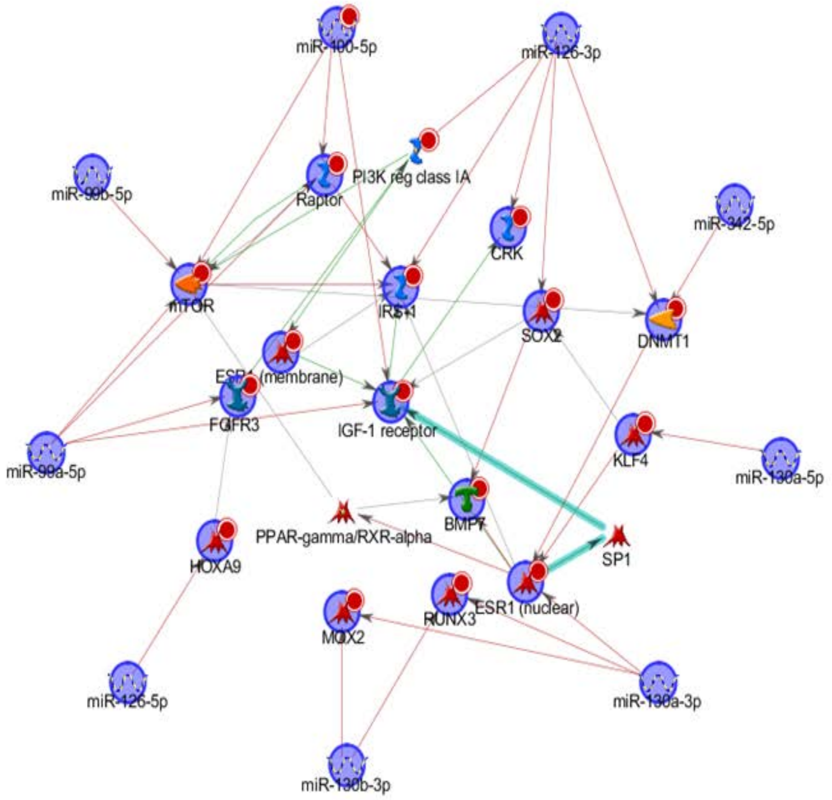

Figure S5. Network of genes regulated by candidate miRNAs (miR-100, miR-99a, miR-99b, miR-342-3p, miR-126, and miR-130a) that were found to be differentially expressed between high- and low-risk IPMNs.
